# The effect of fecal microbiota transplantation on antibiotic-associated diarrhea and its impact on gut microbiota

**DOI:** 10.1186/s12866-024-03261-0

**Published:** 2024-05-09

**Authors:** Le Wang, Gongjing Guo, Yue Xu, Long Li, Bo Yang, Di Zhao, Hongliang Tian, Chen Ye, Zhiliang Lin, Jiaqu Cui, Ning Li, Long Huang, Qiyi Chen

**Affiliations:** 1 Zhangjiagang Hospital affiliated to Soochow University, Suzhou, 215600 Jiangsu Province China; 2 Department of Colorectal Disease, Intestinal Microenvironment Treatment Center, General Surgery of Shanghai Tenth People’s Hospital, Tongji University School of Medicine, Shanghai, 200072 China; 3 Tongji University School of Medicine, Shanghai, 200072 China; 4 Gastroenterology Department of The Second Affiliated Hospital, School of Medicine, The Chinese University of Hong Kong, Shenzhen & Longgang District People’s Hospital of Shenzhen, Shenzhen, 518172 Guangdong Province China

**Keywords:** Antibiotic-associated diarrhea, Fecal microbiota transplantation, Dysbiosis, Efficacy

## Abstract

**Background:**

Antibiotic-associated diarrhea (AAD) refers to symptoms of diarrhea that cannot be explained by other causes after the use of antibiotics. AAD is thought to be caused by a disruption of intestinal ecology due to antibiotics. Fecal Microbiota Transplantation (FMT) is a treatment method that involves transferring microbial communities from the feces of healthy individuals into the patient’s gut.

**Method:**

We selected 23 AAD patients who received FMT treatment in our department. Before FMT, we documented patients’ bowel movement frequency, abdominal symptoms, routine blood tests, and inflammatory markers, and collected fecal samples for 16S rRNA sequencing to observe changes in the intestinal microbiota. Patients’ treatment outcomes were followed up 1 month and 3 months after FMT.

**Results:**

Out of the 23 AAD patients, 19 showed a clinical response to FMT with alleviation of abdominal symptoms. Among them, 82.61% (19/23) experienced relief from diarrhea, 65% (13/20) from abdominal pain, 77.78% (14/18) from abdominal distension, and 57.14% (4/7) from bloody stools within 1 month after FMT. Inflammatory markers IL-8 and CRP significantly decreased after FMT, but there were no noticeable changes in WBC, IL-6, and TNF-α before and after transplantation. After FMT, the abundance of *Bacteroides* and *Faecalibacterium* increased in patients’ fecal samples, while the abundance of *Escherichia-Shigella* and *Veillonella* decreased.

**Conclusion:**

FMT has a certain therapeutic effect on AAD, and can alleviate abdominal symptoms and change the intestinal microbiota of patients.

## Introduction

Antibiotic-associated diarrhea (AAD) refers to diarrhea symptoms that cannot be explained by other reasons after using antibiotics, often accompanied by abdominal pain, bloating, and bloody stools, and is a common complication in antibiotic treatment [1, 2]. The incidence of AAD varies with different antibiotics, and the incidence rate can be as high as 35% in patients receiving specific antibiotic treatment [3]. AAD is thought to be caused by dysbiosis of the gut due to antibiotics, which may lead to overgrowth of specific pathogens and changes in the function of the microbiome [3, 4]. It is estimated that only 15-25% of AAD cases are caused by overgrowth of *Clostridioides difficile* [5]. On the other hand, the occurrence of AAD may be related to the loss of beneficial metabolic activities of intestinal microorganisms. Changes in the composition and quantity of the gut microbiota (even without the overgrowth of pathogenic microorganisms) can lead to disturbances in overall colon metabolism, thereby causing AAD [6, 7]. Colonic microbiota such as *Bacteroides*, *Bifidobacterium*, and *Lactobacillus* can metabolize carbohydrates that the colon cannot absorb as a source of energy, producing short-chain fatty acids (SCFAs) [8]. Antibiotic treatment leading to a reduction in these bacteria can cause increased carbohydrate content in the colon, leading to osmotic diarrhea. In addition, butyric acid is an important energy source for the distal colonic mucosa [9]. A reduction in SCFAs production caused by a decrease in colonic anaerobic bacteria levels can directly cause colonic mucosal dysfunction and diarrhea [10].

Currently, the preventive intervention for AAD is the use of probiotics, which are defined as “live microorganisms that, when administered in adequate amounts, confer a health benefit on the host” [11]. Research has found that probiotics can reduce the overall risk of AAD during and 7 days after antibiotic treatment by restoring homeostasis in the gut [2]. Fecal Microbiota Transplantation (FMT) is a treatment method that involves transplanting the microbiota from a healthy person’s feces into a patient’s gut. This treatment aims to restore balance in the gut microbiota and improve a series of diseases related to the gut microbiota, such as recurrent *Clostridioides difficile* infection (rCDI) [12]. Therefore, research teams have begun to explore the efficacy of FMT on AAD [13]. FMT has been widely used in the treatment of rCDI, with an effective rate of up to 90%. Western countries have already included FMT in the first-line treatment of rCDI [14–17]. This article mainly explores the efficacy and safety of FMT for AAD, and analyzes the changes in the gut microbiota of AAD patients after FMT.

## Materials and methods

### Patient and data collection

This study has been registered on Clinical Trials.gov as the NCT05990972 study. From July 2020 to April 2023, 23 patients with AAD who underwent Fecal Microbiota Transplantation (FMT) in our department were selected. The patient’s information is shown in Table 1. Before FMT, we recorded the patient’s bowel movement frequency, abdominal symptoms, routine blood tests, and inflammation indicators, and collected their stool samples for 16S rRNA sequencing. 1 month and 3 months after FMT, we followed up on the efficacy of the treatment.

**Table 1:** General information of the patients

Characteristics	Overall
Gender (Male/Female)	15/8
Age (mean ± standard deviation)	46.17 ± 16.15
≤50 years	12(52.17%)
>50 years	11(47.83%)
Disease Duration	
≤24 months	11(47.83%)
>24 months	12(52.17%)
Clostridioides difficile test	
Positive	9(39.13%)
Negative	14(60.87%)
Reasons for taking antibiotics	
Digestive system diseases	11(47.83%)
Urinary system diseases	5(21.74%)
Respiratory system diseases	2(8.70%)
Hematological diseases	2(8.70%)
Skin diseases	1(4.35%)
Oral diseases	1(4.35%)
Trauma	1(4.35%)

Inclusion criteria: 1) Clear diagnosis of Antibiotic-associated diarrhea (AAD); 2) Stool samples were preserved during treatment; 3) Complete data on blood routine examination and inflammation factor checks; 4) Ineffective against conventional AAD treatments.

Exclusion criteria: 1) Suffering from other chronic gastrointestinal diseases; 2) History of malignant tumors; 3) History of gastrointestinal surgery; 4) Abnormal colonoscopy findings.

### Results and definitions

Adverse Events (AEs) are recorded during hospitalization for FMT. AEs refer to any new symptoms, worsening of previous symptoms, and abnormal laboratory test results that occur during the FMT process. “Cure” is defined as a defecation frequency of less than or equal to three times per day, complete disappearance of abdominal symptoms, and no recurrence. “Relief” is defined as a defecation frequency of more than three times per day, but less than the frequency at admission, and less severe abdominal symptoms than at admission. Both “cure” and “relief” are collectively defined as a clinical response.

### The donor screening

Donor selection should meet the following criteria: (1) Age between 18 and 30 years; (2) BMI of 18-25 kg/m^2; (3) No pathological signs during physical examination; (4) No history of infectious diseases; (5) No recent gastrointestinal, metabolic, neurological history, or other systemic diseases; (6) No recent use of drugs that could damage the composition of the gut microbiota; (7) Regular healthy diet, appropriate exercise, harmonious family environment, and no smoking or drinking habits; (8) Passing blood and stool tests before donating feces, including general blood and stool tests as well as potential pathogen or infectious disease screenings [18].

### Preparation and procedure of FMT

Approximately 100 g of donated feces is collected into a sterile container, to which 300 mL of saline is added. The mixture is then stirred to allow it to pass through 2.0 mm and 0.5 mm mesh filters. Sterile glycerol is added to reach a final concentration of 10%, and the solution is stored at − 20 °C for 1-8 weeks until use. Patients receive a polyethylene glycol bowel lavage 12-24 hours before FMT. The fecal suspension is thawed in a 37 °C water bath and is infused into the patient through a nasoenteric tube placed in advance, within 6 hours after thawing [18, 19].

### DNA extraction and 16 S rRNA gene sequencing

We used the PowerMax Extraction Kit (MoBio Laboratories, Carlsbad, CA, USA) to extract microbial genomic DNA from fecal samples according to the manufacturer’s instructions. After extraction, we employed agarose gel electrophoresis and NanoDrop ND-1000 spectrophotometer (Thermo Fisher Scientific, Waltham, MA, USA) to measure the concentration and purity of microbial DNA in the fecal samples. To amplify the V4 regions of 16S rRNA, we used two universal primers, specifically 515 Forward Primer (5′-GTGYCAGCMGCCGCGGTAA-3′) and 806 Reversed Primer (5′-GGACTACNVGGGTWTCTAAT-3′). The polymerase chain reaction (PCR) was performed in a 50 μl reaction system, with the PCR cycle consisting of pre-denaturation at 98 °C for 30 seconds, followed by 25 cycles of denaturation at 98 °C for 15 seconds, annealing at 58 °C for 15 seconds, and extension at 72 °C for 15 seconds, with a final extension at 72 °C for 1 minute. Subsequently, we used AMPure XP Beads (Beckman Coulter, Indianapolis, USA) to purify the PCR products and quantified the DNA concentration using the PicoGreen dsDNA Assay Kit (Invitrogen, Carlsbad, CA, USA). After quantitative analysis, the DNA library was sequenced on Illumina NovaSeq 6000 platform with 2 × 250 bp paired-end reads at Shanghai Bao Yuan Pharmaceutical Co., Ltd.

The 16S rRNA sequencing data of all participants in the study has been uploaded to the National Center for Biotechnology Information platform, with the accession number PRJNA1037722.

### Data processing, analysis and visualization

The QIIME2 version 2023.5.0 [20] software was harnessed to implement DADA2 [21] processing on the raw sequencing data. Initially, the data was subjected to quality filtering, effectively eliminating adapter and barcode sequences. The information was also trimmed to an appropriate length to discard sequences that fell below an average quality score of 25. Subsequently, the sequences were dereplicated, scrutinized for sequence variants, merged, and underwent a standard DADA2 procedure for chimera checking. Any amplicon sequence variant (ASV) that had a frequency lower than 50 across all samples or appeared in less than three samples was filtered out. Filtered representative sequences and biom-formatted tables were later assigned using the Greengenes2 2022.10 database. The final result incorporated a table and taxonomy artifacts, which were exported as a biom table and a text file respectively, for future analysis after the addition of taxa data to the biom-formatted ASV table.

The “microeco” package was employed to generate several plots, including Principal Component Analysis (PCA), Principal Coordinate Analysis (PCoA), and Non-Metric Multidimensional Scaling (NMDS) plots based on Jaccard and Unweighted distances, alongside taxonomic composition bar plots, feature abundance box plots, Venn diagrams, and heatmap plots. As the microbiota was represented in relative abundance, Linear Discriminant Analysis (LDA) effect size (LEfSe) analysis [22] was used to observe differences in microbiota composition. After setting the alpha value to 0.05 and the LDA score threshold to 2, the LEfSe bar plot and corresponding cladogram were constructed using the “microeco” package.

The co-occurrence network was calculated using the “microeco” package by employing Spearman analysis for correlation coefficient computation. The *P*-value threshold was set to 0.01, while the coefficient threshold was automatically optimized. Subsequently, the cluster_fast_greedy method was utilized for network clustering, and visualization was conducted using Gephi (v0.10.1).

The Phylogenetic Investigation of Communities by Reconstruction of Unobserved States (PICRUSt2) (v2.5.1) [23] workflow was leveraged to predict the metagenome functions of the microbiota, and functional pathways were annotated using the Kyoto Encyclopedia of Genes and Genomes (KEGG) database [24–27].

### Statistical analysis

In this study, all clinical characteristics are expressed as mean ± standard deviation (SD) or numbers and percentages. The clinical features between different groups were tested using rank sum test and chi-square test. A significance level of *P* < 0.05 was set, and SPSS version 26.0 was used as the statistical tool.

## Results

### Clinical outcomes of FMT treatment for AAD

A total of 23 patients underwent 42 fecal microbiota transplantation (FMT) procedures. Based on the frequency of bowel movements and abdominal symptoms, 82.61% (19/23) of patients responded clinically 1 month after FMT, of which the cure rate was 73.91% (17/23). The frequency of bowel movements significantly decreased. The clinical response rate 3 months after FMT was 78.26% (18/23), with a cure rate of 65.22% (15/23) (Table 2). As shown in Table 3, 65% (13/20) of abdominal pain, 77.78% (14/18) of bloating, and 57.14% (4/7) of bloody stools responded clinically within 1 month after FMT. According to the results of the inflammatory markers and routine blood tests before and after FMT in these 23 patients (Table 4), it was found that the inflammation indicators of AAD patients were higher than normal levels before FMT, and there was a decreasing trend after FMT, especially IL-8 and CRP. However, there was no significant change trend in WBC, IL-6, and TNF-α before and after transplantation.

**Table 2:** Patients clinical outcomes and adverse events following FMT

Pt	Age (year)	CDI	Course	Bowel movement frequency(times/day)		FMT response		AEs
				Pre-FMT	Post-FMT	1 month	3 months	
1	51	P	3	7-8	2-3	Cure	Cure	–
2	57	N	1	5-6	1	Cure	Cure	–
3	23	P	4	10-12	1-2	Cure	Cure	–
4	39	N	1	7-9	2-3	Cure	Cure	–
5	55	P	3	4-5	4-5	Ineffective	Ineffective	Abdominal pain, Abdominal distension
6	65	N	1	4-5	1	Cure	Cure	–
7	21	P	1	4-6	1-2	Cure	Cure	–
8	56	N	1	4-5	1-2	Cure	Cure	–
9	41	N	1	5-6	3-4	Cure	Remission	–
10	28	P	1	3-4	1	Cure	Cure	–
11	23	P	2	5-7	3-4	Remission	Ineffective	Abdominal pain, Fever
12	58	N	2	2-3	1	Cure	Cure	–
13	28	N	2	5-6	2-3	Cure	Cure	–
14	31	P	2	2-3	1-2	Cure	Remission	–
15	69	N	3	3-5	1-2	Cure	Cure	–
16	48	N	3	4-6	1-3	Cure	Cure	–
17	63	N	3	3-5	3-4	Ineffective	Ineffective	Nausea, Vomiting
18	63	N	1	7-8	7-8	Ineffective	Ineffective	Increased frequency of diarrhea
19	37	P	2	3-10	1-2	Cure	Cure	–
20	61	N	1	4-5	3-4	Remission	Remission	–
21	69	N	1	5-6	1	Cure	Cure	–
22	48	N	1	6-8	7-8	Ineffective	Ineffective	Abdominal distension
23	28	P	2	4-6	2-3	Cure	Cure	–

*CDI-P* Possitive, *CDI-N* Negative

**Table 3:** Response to FMT in AAD patients (Units: cases)

	Pre-FMT	Post-FMT	*P*
Diarrhea	23	4^****^	< 0.0001
Abdominal pain	20	7^***^	0.0002
Abdominal distension	18	4^****^	< 0.0001
Hematochezia	7	3	0.2837

**Table 4:** Inflammatory markers before and after FMT

	Pre-FMT	Post-FMT	*P*
WBC(*10^9/L)	6.26 ± 2.22	5.36 ± 1.70	0.2221
IL-6(pg/ml)	18.59 ± 18.99	11.22 ± 13.36	0.2481
IL-8(pg/ml)	171.40 ± 203.33	29.623 ± 31.35^*^	0.0418
CRP(mg/L)	3.48 ± 1.64	1.78 ± 1.31^*^	0.0328
TNF-α(pg/ml)	7.92 ± 7.79	7.29 ± 7.67	0.8444

### The safety of FMT for AAD

**Fig. 1:**
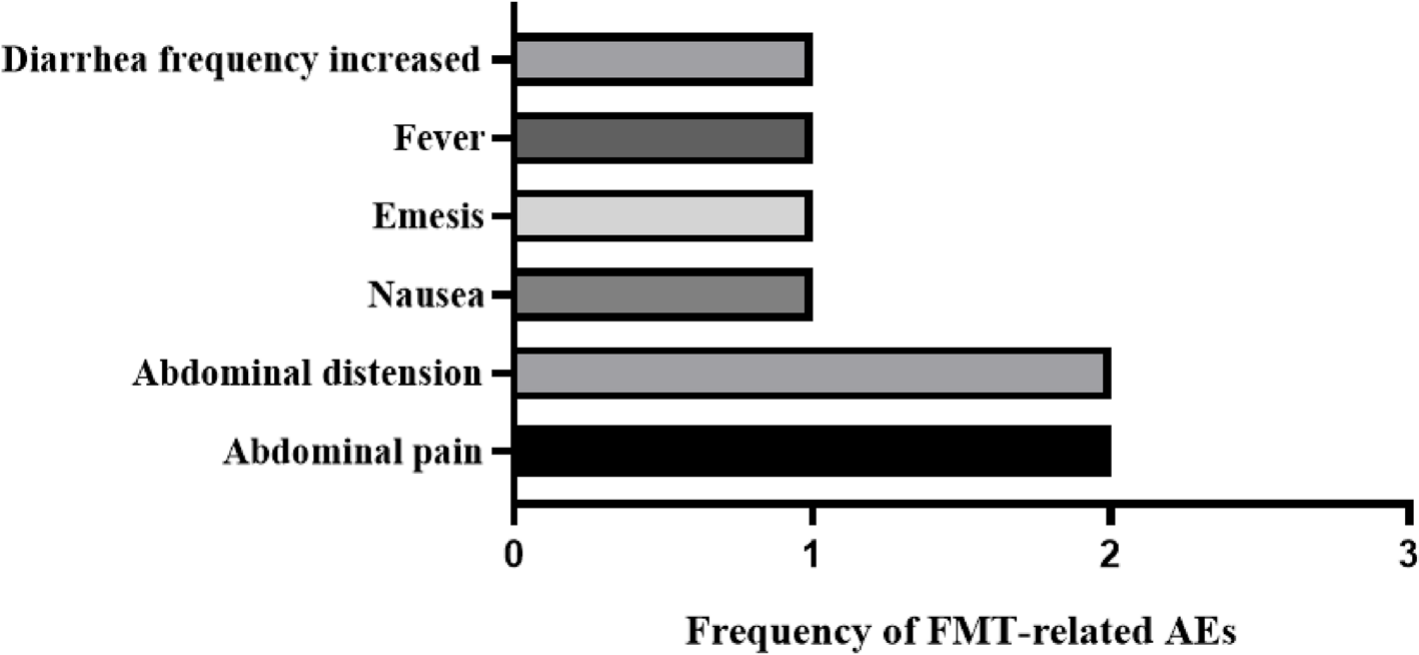
AEs associated with FMT occurring during the FMT period

During the in-hospital FMT period and the three-month follow-up period after FMT, 21.73% (5/23) of patients had eight cases of FMT-related adverse events (AEs) (Fig. 1). The most common AEs were abdominal pain and bloating, each with two cases (2/23, 8.7%), followed by one case (1/23, 4.3%) of nausea, vomiting, fever, and increased frequency of diarrhea. These AEs were all mild, with no FMT-related deaths.

### The changes in the composition and structure of the gut microbiota before and after FMT

**Fig. 2:**
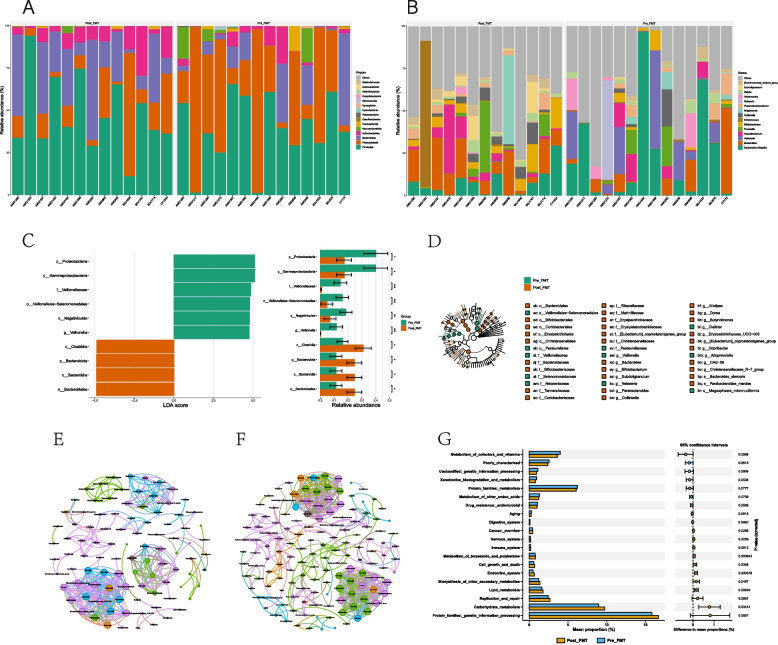
The changes of intestinal microbiota before and after FMT. **A** Phylum level. **B** Genus level. **C** Gut microbiota difference between the before and after FMT were identified with a LEfSe analysis with LDA score threshold> 2.0. **D** The cladogram plot. Co-occurrence network analysis for the before and after FMT. **E** Before FMT. **F** After FMT. **G** KEGG level 2 annotation for the differently abundant function pathway before and after FMT

Figures 2A and 2B illustrate the composition of microbiota at different taxonomic levels. At the phylum level, the top four in terms of abundance after transplantation are Firmicutes, Proteobacteria, Bacteroidota, and Actinobacteriota. Among them, Firmicutes, Bacteroidota, and Actinobacteriota increase after transplantation, while Proteobacteria decreases. At the genus level, the abundance of *Bacteroides* and *Faecalibacterium* increase after transplantation, while the abundance of *Escherichia-Shigella* and *Veillonella* decrease.

Next, we wanted to identify the differences between certain taxonomic groups before and after transplantation, so we conducted a LEfSe analysis, using effect size measurements to enrich bacterial groups with different abundances between the two groups. Under the significance threshold (*p* < 0.05) and LDA score > 2, Fig. 2C shows the taxonomic units of different abundances. We also generated a dendrogram from the LEfSe analysis to provide visual results of the phylogenetic distribution of these samples from the order to genus level. The size of each circle in the cladogram represents the abundance of certain taxonomic groups (Fig. 2D). To further explore the key groups before and after transplantation, we constructed a microbial co-occurrence network (Figs. 2E and 2F) using the Spearman correlation between different groups, where some specific species serve as key nodes in the network. From this network diagram, we can observe that compared to before FMT, after FMT, *Prevotella* and *Bacteroides* are important nodes connected to *Faecalibacterium*. As shown in Fig. 2G, genes related to lipid and carbohydrate metabolism are more enriched after transplantation.

### Differences in the fecal microbiota between clinical responders and non-responders

**Fig. 3:**
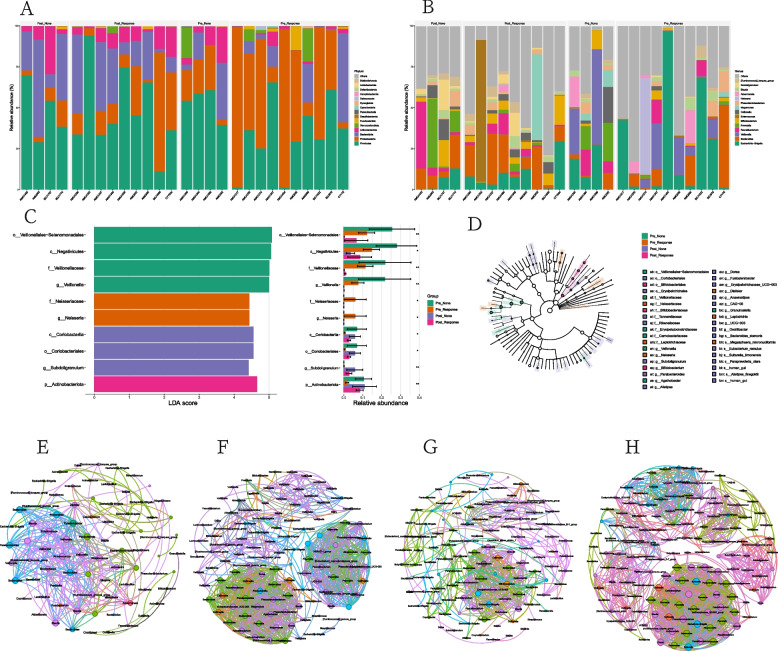
The difference of intestinal microbiota before and after FMT between clinical response group and non-response group. Box plots of microbiota abundance in two groups at different levels before and after FMT. **A** Phylum level. **B** Genus level. **C** Gut microbiota difference between the two groups before and after FMT were identified with a LEfSe analysis with LDA score threshold> 2.0. **D** The cladogram plot. Co-occurrence network analysis for the two groups before and after FMT. **E** Response group before FMT. **F** Non-response group before FMT. **G** Response group after FMT. **H** Non-response group after FMT

Based on the efficacy after FMT, clinical response and non-response groups are divided. Figures 3A and 3B show the microbial composition at different classification levels. At the phylum level, compared with the non-response group, the response group had a lower abundance of Firmicutes and a higher abundance of Proteobacteria before transplantation, and the abundance of Firmicutes increased significantly and Proteobacteria decreased significantly after transplantation. At the genus level, compared with the non-response group, the response group had a higher abundance of *Bacteroides* and a lower abundance of *Prevotella* and *Collinsella* before transplantation. Figures 3C and 3D show the differences in some taxa between the two groups before and after transplantation at the genus level. There are significant differences in *Veillonella*, *Neisseria*, and *Subdoligranulum*. Using the Spearman correlation between different taxa, the co-occurrence networks of the four groups of microbes were constructed. They showed different microbial aggregation networks in different groups. Before and after FMT, the microbial interactions in the clinical non-response group were more closely related (Figs. 3E-3H). Among them, in the pre-FMT response group, *Bacteroides*, *Veillonella*, and *Streptococcus* are important nodes (Fig. 3E), while in the non-response group, *Bacteroides*, *Megamonas*, and *Prevotella* are important nodes (Fig. 3F). After FMT, *Bifidobacterium* and *Fusobacterium* are important nodes in the response group (Fig. 3G), while *Prevotella*, *Paraprevotella*, and *Bacteroides* are important nodes in the non-response group (Fig. 3H).

## Discussion

In this study, 9 out of the 23 patients included were positive for CDI, which accounts for about 15-25% of AAD cases [5]. This is the most widely studied type in AAD [4]. There has been less research on other types of pathogens, which is why the clinical efficacy of FMT for AAD is lower than for CDI. However, in this study, whether there is a CDI infection does not affect the clinical efficacy of FMT for AAD. The clinical response rate for AAD patients who tested positive for CDI was 77.78% (7/9), and the clinical response rate for AAD patients who tested negative for CDI was 78.57% (11/14).

After FMT, we found significant changes in the abundance and diversity of the fecal microbiota, which has a certain correlation with the therapeutic effect of the disease. Studies have found a correlation between improvement in clinical efficacy and changes in the bacterial spectrum after FMT in patients [28, 29]. In rCDI patients who responded clinically, after FMT, there was an enrichment of Ruminococcaceae and Lachnospiraceae, a decrease in Enterobacteriaceae, more obvious colonization of donor microbiota, and alleviation of gut microbiota dysbiosis [30]. In IBS patients post-FMT, gastrointestinal symptom scores (IBS-SSS) decreased, quality of life scores (IBS-QOL) increased, dysbiosis index decreased, and 6 bacteria *Alistipes, Bacteroides spp*, *Prevotella spp*, *parabobacteroides johnsonii*, *Firmicutes*, *Eubacterium biformme*, and *Faecalibacterium prausnitii*, which were negatively correlated with the total IBS-SSS score, were identified [29]. It was also found that the changes in intestinal microbiota after FMT can predict clinical efficacy. Studies have found an index constructed based on *Esherichia* and *Blautia* after FMT can successfully predict the clinical outcomes of rCDI 8 weeks after treatment [30]. Changes in the relative abundance of *Veillonella*, *Ruminococcaceae*, *Eggerthella* and *Lactobacillus*, etc. in the short term (5 days) after treatment may be able to predict the long-term therapeutic effect of FMT on ulcerative colitis (UC) [31].

After FMT, at the genus level, *Veillonella* showed a significant reduction. *Veillonella* is a Gram-negative anaerobic coccus that can produce LPS, inducing an inflammatory response, and it is commonly seen in upper respiratory and intestinal infections. *Veillonella* and *Streptococcus* interact immunologically and often co-occur in ecosystems, suppressing the production of IL-12p70 while enhancing the response of IL-8, IL-6, IL-10, and TNF-α [32]. A study found that compared with the gut microbiota of healthy people, patients with autoimmune hepatitis (AIH) have reduced diversity and enriched genera such as *Streptococcus* and *Veillonella* [33]. In this study, the overall inflammation level of AAD patients after FMT showed a decreasing trend, which was negatively correlated with the changes in *Veillonella*. This is consistent with reports from other studies.

After FMT, the non-responsive group had a higher *Sutturella timonensis* than the responsive group. Some studies have found that *Sutterella* may play an important role in FMT treatment responses, where the abundance of *Sutterella* is negatively correlated with the remission degree of Ulcerative Colitis (UC) [34]; and in clinical cohort studies, it was found that the abundance of *Sutterella* is negatively correlated with the level of inflammatory cytokines (IL-12, IL-13, IFN-γ) [35]; in related experiments on AAD, high-throughput sequencing of fecal samples from 30 seven-week-old SPF male rats revealed that the relative abundance of the genus *Sutterella* is negatively correlated with the dose and positively correlated with the development of AAD [36]. At the same time, we found that the composition of the gut microbiota of patients before FMT is one of the main factors affecting clinical outcomes, which has been proven by numerous clinical studies [14]. For instance, in ulcerative colitis, higher fecal and mucosal microbial abundance before FMT treatment is associated with positive treatment outcomes, while the abundance of *Clostridium* and *Sutterella* is associated with FMT treatment failure [14, 34]. In Crohn’s disease, FMT failure is associated with the enrichment of different γ-Proteobacteria members (such as *Klebsiella*, *Actinomyces*, and *Hemophilus*) in the baseline recipient microbiota [37]. IBS patients who responded to FMT had a higher baseline abundance of streptococcal species and higher microbial diversity than non-responsive patients [38].

The limitations of this study include a small sample size and lack of controls. The fecal microbiota was only assessed using 16S rRNA sequencing, and there is a lack of related metabolomics data, so changes in metabolites related to short-chain fatty acids, bile acids, and lipids before and after FMT cannot be compared. Comprehensive microbial analysis may further deepen the understanding of the potential of precise strains and their specific functions as predictive factors for FMT success and failure. Therefore, we plan to increase the number of samples in subsequent studies, and perform metagenomic sequencing and related metabolomics sequencing on fecal samples.

## Data Availability

The 16S rRNA sequencing data of all participants in the study has been uploaded to the National Center for Biotechnology Information platform, with the accession number PRJNA1037722.
